# Qualitative Health-Related Quality of Life and Natural Language Processing: Characteristics, Implications, and Challenges

**DOI:** 10.3390/healthcare12192008

**Published:** 2024-10-08

**Authors:** Esther Lázaro, Vanessa Moscardó

**Affiliations:** Faculty of Health Sciences, Valencian International University, Calle Pintor Sorolla 21, 46002 Valencia, Spain; vanessa.moscardo@professor.universidadviu.com

**Keywords:** health-related quality of life, quality of life, natural language processing, large language models

## Abstract

Objectives: This article focuses on describing the main characteristics of the application of NLP in the qualitative assessment of quality of life, as well as its implications and challenges. Methods: The qualitative methodology allows analysing patient comments in unstructured free text and obtaining valuable information through manual analysis of these data. However, large amounts of data are a healthcare challenge since it would require a high number of staff and time resources that are not available in most healthcare organizations. Results: One potential solution to mitigate the resource constraints of qualitative analysis is the use of machine learning and artificial intelligence, specifically methodologies based on natural language processing.

## 1. Introduction

Over recent decades, there has been an increasing prevalence of chronic disorders, causing a deterioration in the quality of life of patients. It is very important that the different people involved in their care have tools to evaluate, measure, and report the experience and health outcomes of patients in order to improve their quality of life. In recent years, there has been a renewed effort focused on evaluating Patient-Reported Outcomes and Experience Measures (PREMs/PROMs). This demonstrates the importance of integrating the patient perceptions, needs, and quality of life in care delivery.

Quality of life is defined as the general satisfaction and well-being in a specific environment across different domains [1,2,3]. Health-related Quality of life (HRQoL) can be defined as how well a person functions in their life and their perceived well-being in physical, mental, and social domains of health [4]. World Health Organization (WHO) defines quality of life as an individual’s perception of their position in life in the context of the culture and value systems in which they live and in relation to their goals, expectations, standards, and concerns [5]. Although these terms are widely used in the literature, a debate remains about the definition of these concepts [3].

Health-related quality of life can be measured objectively using quantitative data and information about the living environment and, also subjectively, by evaluating the levels of satisfaction and narratives that people feel about their quality of life. There has also been increasing interest in combining both approaches [6]. The classic procedure involves the use of numerical scales, for example, the Likert scale, to rate patients’ perceptions and experiences. Qualitative data can be collected in the form of stories or comments, often through unstructured free text. This approach provides valuable information and can complement quantitative data. These free text comments should be collected and analysed with the same scientific rigour as the quantitative approach [7,8,9,10].

The in-depth qualitative methodology facilitates the evaluation of the areas that affect health-related quality of life in relation to a specific pathology and the problems associated with each of them. Furthermore, given the evolution and possible degeneration of health in the disease process, it may be necessary to assess the change in quality of life in order to obtain a longitudinal, valid, and sensitive assessment. Although the physical symptoms may be common to patients, the experience linked to the disease often differs. Furthermore, in the same group of pathologies, there are patients who may experience a different experience derived from the severity of the symptoms and the evolution of the individual process. This implies the effort of developing and validating a quality-of-life questionnaire for each pathology and also taking into account the complex profile of the chronic patient or the multi-pathological patient [11,12].

Among the advantages offered by the application of qualitative research methods, it is worth highlighting the possibility of obtaining a detailed description of the feelings, opinions, and internal experiences of the participants using a flexible and open approach. In addition, it tries to take into account the experience of patients, in a holistic way, in specific environments. As a result, complex issues, such as health-related quality of life in chronicity processes, can be more easily understood [13].

The qualitative methodology allows analysing patient comments in unstructured free text and obtaining valuable information through manual analysis of these data. However, for most healthcare organizations, collecting large volumes of data are a challenge that requires investments of time and personnel that are often not available. This process would be facilitated if there were a systematic way to extract useful information from patients’ free-text comments. This focus will improve the quality of care for patients with chronic diseases [7,8,9,10].

One potential solution to mitigate the resource constraints of qualitative analysis is the use of machine learning and artificial intelligence, specifically methodologies based on natural language processing. Natural language processing and machine learning are critical tools for processing unstructured free text. Artificial intelligence applied to text data is based on the use of computer algorithms that handle, augment, and transform natural language so that it can be processed for computation. Natural language processing is used to extract information, convert unstructured text into a structured format, perform syntactic processing, capture meaning, and identify relationships between concepts. This text processing considers the use of defined language rules and relevant domain knowledge. Currently, the big data analytics techniques and artificial intelligence are widely used in healthcare [14,15,16,17,18,19,20].

Another approach to deal with text data and extract information about its content and context is the use of large language models (LLMs). LLMs learn statistical relationships from text documents during a computationally intensive self-supervised and semi-supervised training process [21]. LLMs are artificial neural networks, the largest and most capable of which are built with a transformer-based architecture. The pioneer and the first relevant model that used this technology was the Bert model, which was introduced in 2018 [22].

Other notable LLMs and recent ones are OpenAI‘s GPT series of models (e.g., GPT-3.5 and GPT-4, used in ChatGPT and Microsoft Copilot) [23], Google‘s PaLM and Gemini (used in Bard) [24], and Meta‘s LLaMA family of open-source models [25]. These are constant improving and evolving.

This article contributes to the literature by focusing on new valid and reliable tools for the qualitative assessment of the quality of life of patients with chronic diseases based on the potential of artificial intelligence and natural language processing.

## 2. Characteristics

Table 1 presents a comparison between qualitative and quantitative methods of quality-of-life assessment analysis, as well as the advantages of natural language processing by AI solutions. As can be seen in this table, the analysis of health-related quality of life, through natural language processing, offers characteristics that can offer great potential in the field of health research.

### 2.1. Dimension of Group Studies

On the one hand, it offers the advantage of working with a large amount of qualitative data from large samples of patients and pathologies in different phases and health processes. The use of trained, reliable, and validated algorithms would help the clinician analyse a large amount of data with less effort and time. The algorithm used for this purpose will be the basis for the advanced application of qualitative data categorization and the obtention of quality-of-life analysis reports. These will be able to estimate which categories correspond to each fragment of the texts of the evaluated patients through text processing and text mining.

### 2.2. Understand the Context of the Problem and Scope of the Study in Terms of Time

This perspective can help understand the context and experience of the patient not only at a specific moment in their experience but, longitudinally, over several moments and contexts that the researcher considers necessary, for example, at the time of diagnosis, during the treatment to analyse qualitative data about the perception regarding treatment adherence and satisfaction with doctor–patient communication, etc. This perspective would be very useful not only to have knowledge of the health-related quality of life as perceived by the patient, but also it can be expanded and complemented with information from the immediate environment, including family members and health professionals. Likewise, the methodology provides a large amount of valid and reliable information that can support the effectiveness of the implementation of multidisciplinary intervention programs with chronic pathologies. Furthermore, the methodology would be applicable to both data written by the patient himself or clinicians through notes and clinical records and data collected orally through interviews and their transcriptions.

### 2.3. The Researcher’s Point of View

NLP analyses texts and narratives that contain the direct experience of patients. The design and development of models based on NLP involve a prior training phase in which the participation of clinical experts is essential. This phase refers to the semantic analysis of the conversations and annotations made by clinical psychologists in order to generate labels, in the form of keywords, that refer to their contents. These labels could also refer to health-related quality of life factors. Subsequently, the developed AI systems will be trained to be able to classify and automatically label the fragments of the patients’ narratives with a certain precision. Then, the conversations and interviews will be automatically classified according to the categories proposed by psychology experts, which have been established at the time of the study design. In this way, the algorithm goes through a training process based on concurrent validation by clinical experts.

### 2.4. Theoretical Framework and Hypothesis

Natural language processing can be applied to the different methodologies offered by qualitative research, including focus groups, case studies, structured interviews, in-depth interviews, or observational studies. In all of them, texts and narratives are collected, and they can be analysed through techniques based on AI algorithms and NLP.

The application of the deductive thematic analysis methodology to texts and narratives, through training with NLP techniques, would provide a representative number of factors and components of health-related quality of life that would encompass the experience of the entire of chronic pathologies. The deductive approach is initially based on the factors of conceptualizations, theories, tests, and questionnaires already validated based on health-related quality of life, and it can be expanded with new information from patients. The most relevant factors found in the literature are the ones that generally cover dimensions such as physical function, mobility, cognitive challenge, social, emotional, satisfaction with life, work, educational, and economic, among others. The flexible framework of NLP allows new factors to be included as they arise (for example, the impact on quality of life in relation to telemedicine).

### 2.5. Design and Development of Models Based on NLP and Artificial Intelligence

Quality-of-life analysis through natural language processing involves different phases. The first phase requires the semantic analysis of the conversations and annotations made by clinical psychologists in order to generate labels, in the form of keywords, that refer to their content. For automatic labelling, AI systems can be implemented that use various innovative technologies such as machine learning techniques for the automatic extraction of relevant fragments or segments of the conversations. Artificial intelligence technologies applied to natural language processing can also be applied, specifically, speech-to-text methods for the transcription of voice from recordings and text mining for the processing and lemmatization (extraction of root elements) of the transcribed content and the relevant fragments extracted in the previous point.

From the labels generated in the analysis, the conversations and interviews will be automatically classified according to the categories proposed by the expert psychologists, which have been established at the time of the study design.

The algorithm used for this purpose will be the basis for the advanced application of interview categorization reports and quality-of-life analysis. Using text processing and mining, it will be possible to estimate which categories each patient belongs to based on the characteristics obtained in the previous points. To achieve this objective, the implementation of a functional architecture is proposed in two layers:

Layer 1. semantic analysis: it is a layer of semantic analysis and automatic generation of metadata from conversations and interviews. The purpose is automatic labelling so that keywords associated with them can be generated. This analysis will result in the following sources of information:

Set of content tags (mainly contextual information) generated from the analysis of conversations and interviews. Specifically, textual information is stored as phrases and words present in the audio, as well as the analysis of the interviews and notes of the expert and other metadata associated with each patient. The information regarding the audio recordings will be obtained through specialized software based on AI for the automatic transcription of videos. This software is characterized by integrating automatic voice recognition tools as well as functionalities that allow easy integration with other machine learning (ML) toolkits, since they usually offer interfaces in the Python 3.13.0rc3 programming language, such as TensorFlow 2.17 or PyTorch 2.4.

The text of the transcripts and notes will be processed using text mining techniques using natural language processing. Since the texts are expected to be long and unwieldy, the first functionality to be implemented will be the automatic summary of the text to reduce the size and keep only the tokens or words that have more content and are representative.

Taking these labels into account, keyword extraction will be performed. To do this, KeyBERT will be used, a minimal and easy-to-use keyword extraction technique that takes advantage of BERT embeddings to create the keywords and phrases closest to a document. In addition, the use of cosine similarity will allow subphrases to be found in the document that are most appropriate to the document itself and therefore the best to describe it.

Layer 2. categorization, estimation of quality-of-life categories: On this first layer, the deployment of an advanced layer of applications and services is proposed that provides a categorization of patients based on the previously extracted metadata, according to the different proposed classifications. In this way, each patient will have associated quality-of-life categories.

At a technical level, to obtain the categorization, this system will explore approximations of models based on clustering, semantic clustering, and semantically enriched rules, as well as neural networks or a combination of these. After the evaluation and validation of the proposed architectures and designs, the models with the best performance will be established as final classification models.

### 2.6. Validation Processes

Once the algorithms have been implemented and the flow of this AI module has been configured, the automatic labelling and classification system must be validated by three actions:

- Cross-validation of the proposed trained classification models, in particular by 4-fold validation: The division of the dataset into training and test data will be 80–20%.

The metric to evaluate the goodness of fit of the model can be established by analysing the ROC curves of the identified models, considering that the output of these models is multiclass. In this analysis, the sensitivity and specificity of the models can be assessed.

- Estimation of the efficiency of the final model: The efficiency of the model will be defined in terms of the time needed by the algorithm to perform the training (training time) and obtain the results (prediction time). In addition, the complexity of the model will be quantified by defining the orders of the proposed models.
- Implementation of a demo in which it will be possible to review and check the processes carried out in each task of this module until obtaining the categorization results.

### 2.7. Clinical Applications

New approaches are required to complement and go beyond evidence-based medicine in the field of chronic diseases [26]. There are several studies where data collected through social media to assess subjective well-being and happiness by collecting and analysing messages [27,28]. Other sources of information for natural language analysis are physicians’ visit notes in electronic medical records. Electronic medical records and additional patient data are analysed for clinical and translational research. Machine learning-based methods for processing electronic medical records are resulting in a better understanding of patient status and prediction of chronic disease risk. This provides the opportunity to gain previously unknown clinical insights. However, a large number of stories remain inaccessible due to free-text clinical narratives. The review by Sheikhalishashi’s team [26] provides a comprehensive overview of the development and adoption of natural language processing methods applied to free-text clinical notes related to chronic diseases.

Another article analyses qualitative information from electronic medical records on the symptoms of patients with cancer in a free-text format [9]. The aim of that study was to create machine learning algorithms capable of extracting symptoms reported by patients from medical record notes. The procedure included manually annotating 10,000 sentences and then training a conditional random field model to predict words indicating an active symptom (positive label), absence of a symptom (negative label), or no symptom (neutral label). The authors highlight that, although symptoms are important for quality of life and often lead to changes in clinical status, computational methods to assess symptom changes over time are not developed.

Natural language processing techniques have been studied and applied to multiple health areas in recent years, but there are few studies focused on the assessment of quality of life. Other study [28] explored the use of social media in quality of life, capturing and mapping people’s perceptions of their lives based on social media. The methodology is based on a mixed-method approach combining manual message coding, automated classification, and spatial classification. They concluded that data obtained through social media could be used to assess quality of life as a complement to quality-of-life surveys.

NLP has also been used to assess the quality of palliative care and end-of-life care in children with serious illnesses, such as childhood cancer. The findings of the study indicate that NLP is a viable method for measuring the quality of end-of-life care for children with cancer and is a suitable method for multi-centre research and quality improvement [17,29].

## 3. Implications

The analysis of health-related quality of life through NLP or LLM are instruments applicable to people with chronic diseases. This specific target group represents a large percentage of world population and a relevant public health problem. One of the main implications is the development of a tool to support and complement the current quantitative evaluations carried out with specific questionnaires on quality-of-life or classic qualitative analyses. As mentioned above, it would represent a great saving of time in the analysis of quality of life applicable to all chronic pathologies, including rare diseases that usually receive less attention in the research environment. For instance, this automated methodology will generate valid and reliable data on health-related quality of life, and it will serve as a methodology for the analysis of patient narratives, including interviews and clinical histories, whether in a written or oral format.

In the socio-health field, in both clinical practice and research, this tool would facilitate the evaluation of intervention programs of the most diverse nature, as well as the costs associated with the treatment of chronic diseases based on their perception of quality of life. Furthermore, the nature of this tool would promote the transference of the methodology in different contexts, including the socio-health field, hospitals, universities, research centres, medical industry, or patient associations.

In addition, it would mean cost savings in the evaluation of quality of life based on qualitative techniques and the cost associated with the development of specific tools for each rare and chronic disease would be strongly reduced since the tool will be independent of the diseases. This would mean an increase in the number of people and groups susceptible to being evaluated in their subjective quality of life, in a rigorous, reliable, in-depth, and more complete way, compared to quality-of-life measures, and more limited in depth, based on scales and tests.

Then, Patient-Reported Outcome Measures (PROMs) would be strengthened, specifically those focused on quality of life, from the point of view of clinical validity and efficiency. This is crucial to value people’s quality of life as the backbone of health and promote people’s well-being, considering health-related quality of life as a fundamental aspect in the person-centred healthcare strategy.

## 4. Challenges

The current trend in methodologies for evaluating quality of life are focused almost exclusively on classic quantitative and qualitative evaluation models. Thus, an approach based on AI algorithms and NLP techniques for the evaluation of quality of life related to health becomes relevant and poses challenges and uncertainties that must be considered.

In this sense, a research area is created to develop this methodology. To this end, it is important to start with a huge and updated amount of data that can be analysed by trained personnel and, subsequently, categorized into classic factors associated with quality of life related to health. This categorization, carried out by trained clinicians and health experts, will be the basis for the subsequent training of the algorithm and constant updating of the system. The system will always consider the classic psychometric criteria of reliability and validity typical of the development of quality assessment tests. Another challenge of this methodological perspective is the accessibility to samples and personal data. This fact highlights the need to have open repositories and databases so that researchers can access them, considering the anonymity of the participants.

Any dataset used to train AI systems requires careful handling, especially in the context of natural language processing (NLP). An important point to highlight is the great variety of expressions within the same category. This creates challenges in semantic classification within NLP. Expressions are not always uniform, and the same concept or sentiment can be expressed with different words, idioms, or grammatical structures. To do so, AI needs to be trained with a wide range of examples that cover these variations. It can also happen that the same word or expression can belong to different categories depending on the context. The influence of language and cultural connotations are another factor to take into account since linguistic expressions are deeply linked to the language and the cultural context in which they are used. A model trained exclusively in one language may not correctly capture these subtleties when faced with interviews in other languages. Lastly, context is key to correctly interpreting expressions and categorizing them appropriately. AI models need to be able to interpret not only the literal meaning of words but also the nuances of the context in which they are used. This can be particularly difficult in interviews, where the tone, intent, and emotion behind the words also play a crucial role.

It is necessary to continue developing natural language processing techniques for qualitative texts, obtaining models and implementing artificial intelligence techniques aimed at increasing information on the health and experience of patients with chronic diseases. Although there are exploratory studies in this line, there is no validated tool based on NLP that evaluates the quality of life in chronic pathologies from free text, making future research focused on the comparison between traditional qualitative evaluation techniques and methodologies based on NLP. These efforts should be directed towards the creation of an automated analysis tool for the quality of life in chronic diseases based on NLP and AI [9,17,26,28,30].

**Figure 1:**
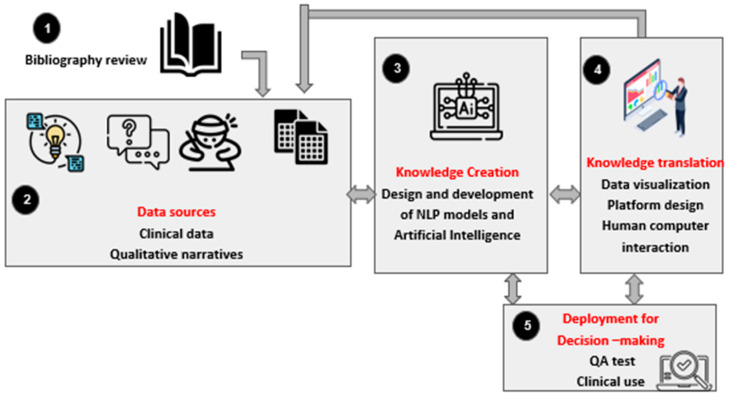
Phases in the process of developing the NLP-QoL tool. 1. Bibliography review; 2. Data sources; 3. Knowledge creation; 4. Knowledge translation; 5. Deployment for making-decision.

Figure 1 shows the main phases in the process for developing the NLP-QoL tool. The phase 1 corresponds to the generation and collection of data from patients’ narratives, interviews, and notes from professionals. Then, a labelling of all these data is carried out by the experts. The phase 2 is related to the design and development of the AI models, which will be classification models based on NLP and/or LLM techniques. These models will be trained and validated with the data from phase 1. Phase 2 will be finished when an adequate accuracy and precision of the models will be achieved. Phase 3 corresponds to the implementation of the platform interface where the doctors can validate and visualize the results. They will be able to introduce the material related to the narratives from patients and obtain the results and labelling of the text in terms of quality-of-life evaluation. The last phase will be an assessment of the quality tests of the models and the platform. This will allow to have accurate models and a platform with good functioning.

## Figures and Tables

**Table 1:** Comparative between quantitative–qualitative investigation and solutions based on AI.

Dimension	Quantitative Investigation	Qualitative Research	Natural Language Processing
Dimension of group studies	Minor	Higher	Higher
Understand the context of the problem	Minor	Higher	Higher
Proximity of the researcher to the problem	Minor	Higher	Higher
Scope of the study in time	Immediate	Longer range	Longer range
The researcher’s point of view	External	Internal	Internally trained algorithm for clinical concurrent validation
Theoretical framework and hypothesis	Well structured	Less structured	Well structured
